# Current use of echocardiography in cardio-oncology: nationwide real-world data from an ANMCO/SIECVI joint survey

**DOI:** 10.1093/ehjimp/qyae081

**Published:** 2024-08-12

**Authors:** Andrea Barbieri, Massimiliano Camilli, Irma Bisceglia, Francesca Mantovani, Quirino Ciampi, Concetta Zito, Maria Laura Canale, Georgette Khoury, Francesco Antonini-Canterin, Scipione Carerj, Marco Campana, Carmine Riccio, Michele Massimo Gulizia, Massimo Grimaldi, Domenico Gabrielli, Furio Colivicchi, Mauro Pepi, Fabrizio Oliva

**Affiliations:** Cardiology Division, Department of Biomedical, Metabolic and Neural Sciences, University of Modena and Reggio Emilia, Policlinico di Modena, Via del Pozzo, 71, Modena 41124, Italy; Department of Cardiovascular Medicine, Fondazione Policlinico Universitario A. Gemelli IRCCS, L.go A. Gemelli, 1, Rome 00168, Italy; Department of Cardiovascular and Pulmonary Sciences, Catholic University of the Sacred Heart, Rome, Italy; Integrated Cardiology Services, Department of Cardio-Thoracic-Vascular, Azienda Ospedaliera San Camillo Forlanini, Rome, Italy; Cardiology Division, Azienda USL—IRCCS di Reggio Emilia, Reggio Emilia, Italy; Cardiology Division, Fatebenefratelli Hospital, Benevento, Italy; Cardiology Division, University Hospital Polyclinic G. Martino, University of Messina, Messina, Italy; Division of Cardiology, Azienda USL Toscana Nord-Ovest, Versilia Hospital, Lido di Camaiore, Italy; Cardiology Division, A.O. Santa Maria, Terni, Italy; Rehabilitative Cardiology, Rehabilitative Hospital High Speciality, Motta di Livenza, TV, Italy; Cardiology Division, University Hospital Polyclinic G. Martino, University of Messina, Messina, Italy; Cardiology Division, Humanitas Gavazzeni, Bergamo, Italy; Cardiovascular Department, Follow-Up del Paziente Post-Acuto, A.O. Sant'Anna e San Sebastiano, Caserta, Italy; Cardiology Division, Ospedale Garibaldi-Nesima, Azienda di Rilievo Nazionale e Alta Specializzazione ‘Garibaldi’, Catania, Italy; Cardiology Division, Ospedale Miulli, Acquaviva delle Fonti, Bari, Italy; Division of Cardiology, Azienda Ospedaliera San Camillo-Forlanini, Rome, Italy; Department of Clinical and Rehabilitation Cardiology, Ospedale San Filippo Neri, Rome, Italy; Cardiology Division, Centro Cardiologico Monzino, IRCCS, Milan, Italy; Cardiologia 1 - Emodinamica Dipartimento Cardiotoracovascolare ‘A. De Gasperis’, ASST Grande Ospedale Metropolitano Niguarda, Milan, Italy

**Keywords:** cardio-oncology, echocardiography, advanced echocardiography techniques, national survey

## Abstract

**Aims:**

The need for cardio-oncology competencies is constantly growing, and with the establishment of cardio-oncology services, cardiovascular imaging, particularly transthoracic echocardiography (TTE), has become pivotal in patients’ management. However, care pathways for oncologic patients largely depend on local health structures’ resources. This survey from Associazione Italiana Medici Cardiologi Ospedalieri and the Italian Society of Echocardiography and Cardiovascular Imaging aimed at investigating the use of echocardiography in cardio-oncology services and knowledge levels on cancer patients’ care.

**Methods and results:**

Data were obtained via an electronic survey based on a structured questionnaire uploaded to the promoting societies’ websites. Responses came from 159 centres with echocardiography. According to one-third of participating centres, workload related to cancer patients represented >30% of the total requests. The most common TTE indication (85%) was left ventricular ejection fraction (LVEF) evaluation. Many centres (55%) still assessed LVEF solely by bidimensional method or visual estimation in case of inadequate acoustic windows. At the same time, almost 40% of centres reported routinely using global longitudinal strain when feasible. We further performed a sub-analysis according to the presence (33%) or absence (77%) of dedicated cardio-oncologists, revealing significant differences in cardiovascular surveillance strategies and cardiotoxicity management.

**Conclusion:**

This survey on echocardiography practice for cancer patients reveals a significant gap between actual clinical practice and standards proposed by recommendations, underlying the need for stronger partnerships between cardiologists and oncologists and dedicated, well-structured cardio-oncology services.

## Graphical Abstract

**Graphical Abstract:**
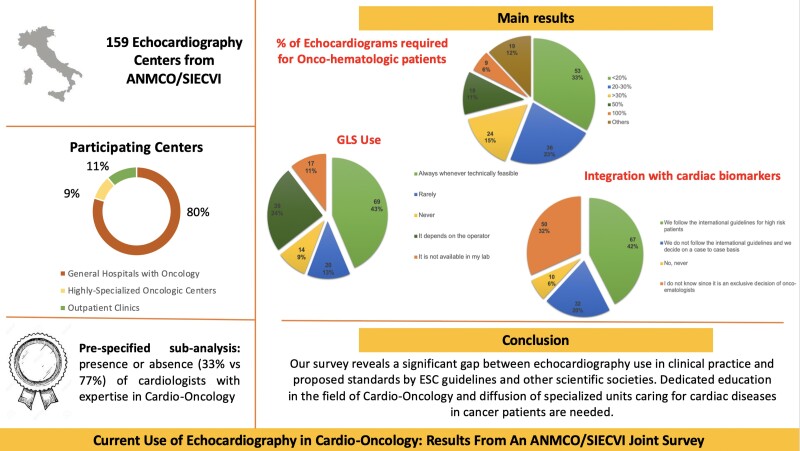
The main information on participating centres, sub-analysis performed, and results obtained. ANMCO, Associazione Nazionale Medici Cardiologi Ospedalieri; ESC, European Society of Cardiology; GLS, global longitudinal strain; SIECVI, Società Italiana di Ecocardiografia e CardioVascular Imaging.

## Introduction

The need for cardio-oncology services is constantly growing^1,2^ due to the rising prevalence^3^ and prognostic impact of cardiovascular diseases in cancer survivors,^4–6^ as well as the growing complexity of current anticancer therapies associated with a plethora of cardiac side effects.^7^ Anthracyclines’ cardiotoxicity represents the paradigm on which cardio-oncology builds. These compounds may be mainly responsible for cardiac dysfunction, eventually leading to overt heart failure if associated with concomitant cardiotoxic therapies (e.g. Human Epidermal Growth Factor Receptor 2, HER2 inhibitors). In this context, a significant decline in left ventricular performance has been described in up to 8% of exposed patients. In comparison, subclinical changes in myocardial function could reach an incidence of almost 20%.^8^ Transthoracic echocardiography (TTE) represents the most accessible imaging technique in this field and is primarily recognized as a fundamental tool in cancer patients’ management.^9^ Accordingly, the European Society of Cardiology (ESC) recently defined the Core Curriculum requirements for providing standardized and harmonized cardio-oncology care, recognizing among fundamental skills of cardio-oncology specialists the interpretation of cardiovascular imaging findings. At the same time, cancer patients’ care should be shared between oncologists, cardio-oncology specialists, and imaging experts through a multidisciplinary approach.^2^ However, in everyday practice, the management of this population largely depends on the organization of local health structures,^10^ and decentralized studies have resulted in the lack of agreement on the best cardiac imaging applications in modern cancer therapy settings.^11^ This is the main reason for a joint research effort made by the Associazione Italiana Medici Cardiologi Ospedalieri (ANMCO) and the Italian Society of Echocardiography and Cardiovascular Imaging (SIECVI), aimed at understanding the routine workload required for oncologic patients in terms of echocardiography activity and current application of echocardiographic modalities in this population in Italy. In detail, methods of heart function estimation and concomitant use of cardiac biomarkers were investigated. Real-world data might offer insights into how health systems and clinical settings can influence cardio-oncology practice.^12^ This information might help build next-generation cardio-oncology services and affect future strategies to optimize the integration of cardiovascular imaging modalities into regular patient evaluation.

## Methods

We analysed the use of cardiac imaging, focusing on echocardiography, in cancer patients treated with potentially cardiotoxic therapies in Italy. This national survey was available for 3 months (March–May 2023). Data were retrieved via an electronic survey based on a structured questionnaire uploaded on the ANMCO (www.anmco.it) and SIECVI (www.siecvi.it) websites. Each centre belonging to both societies was contacted separately by email. The methodology of the surveys has already been described.^13–15^ The questionnaire required general information for each interviewed physician, including working hospitals and departments, cities, and regions in Italy. In summary, participants were asked the following queries: (i) the kind of hospital organization in which they worked; (ii) the number of TTE performed in cancer patients; (iii) the presence of cardiologists dedicated to this specific activity; (iv) the principal indication for TTE in cancer patients; (v) how often TTE was required for patients undergoing anthracycline and trastuzumab therapy; (vi) use of global longitudinal strain (GLS); (vii) use of the left atrial strain; (viii) use of ultrasound contrast agents (UCAs) during echocardiography; (ix) the echocardiographic modality of evaluation and analysis of left ventricular ejection fraction (LVEF); and (x) concomitant use of serum biomarkers during anthracycline and trastuzumab therapy. A complete list of questions submitted to cardiology centres relying on echocardiography is presented in Supplementary data online, *File S1*.

Only one respondent was selected for each participating centre; completed questionnaires were double-checked to avoid duplicates from the same institution.

### Statistical analysis

Categorical data are expressed in terms of the number of subjects and percentage, while continuous data are expressed as mean ± standard deviation or median (minimum–maximum) depending on the variables’ distribution. The *χ*^2^ test or Fisher exact test was used to compare the distribution of categorical variables among groups. Statistical analysis was performed using the JMP PRO software package, version 16 (SAS Institute Inc., Cary, NC).

## Results

**Figure 1:**
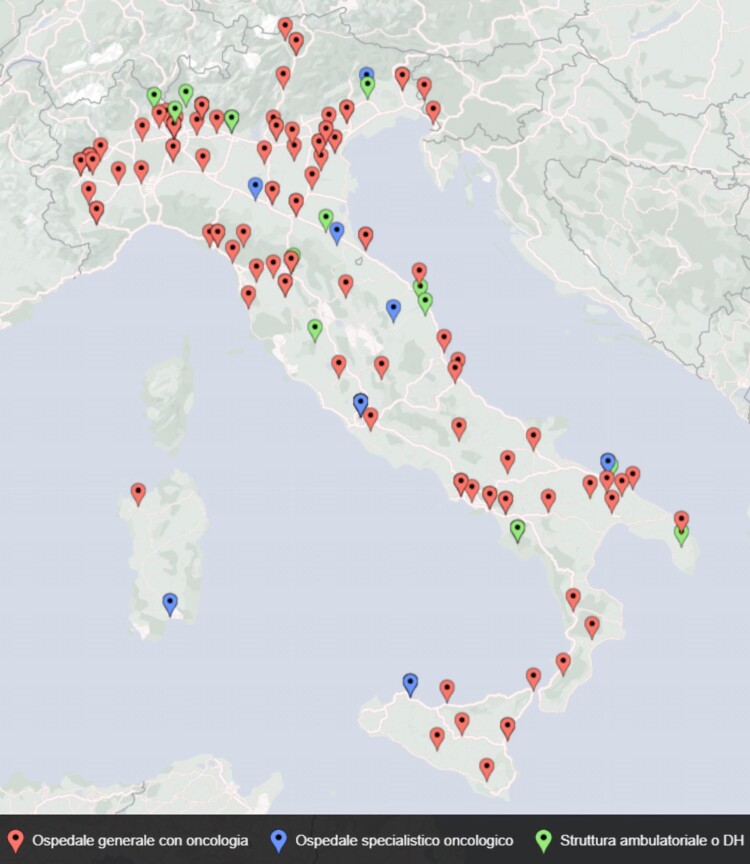
Visual localization of participating centres to the joint ANMCO/SIECVI survey on cardio-oncology imaging practice in Italy. Red bullets stand for general hospitals, blue for highly specialized oncologic hospitals, and green for cardiology outpatient services.

The main data from the participants and survey results are sketched in the *Graphical Abstract*. Data were obtained from 159 echocardiographic laboratories: 84 centres (53%) were in the northern regions of Italy, 31 centres (19%) were in the central, and 44 (28%) were in the southern regions. Participating centres are visually presented in *Figure 1*. Participating centres were divided into 127 (80%) general hospitals with an oncology division, 14 (9%) highly specialized oncology hospitals, and 18 (11%) outpatient clinics with cancer patients referred from other centres.

**Figure 2:**
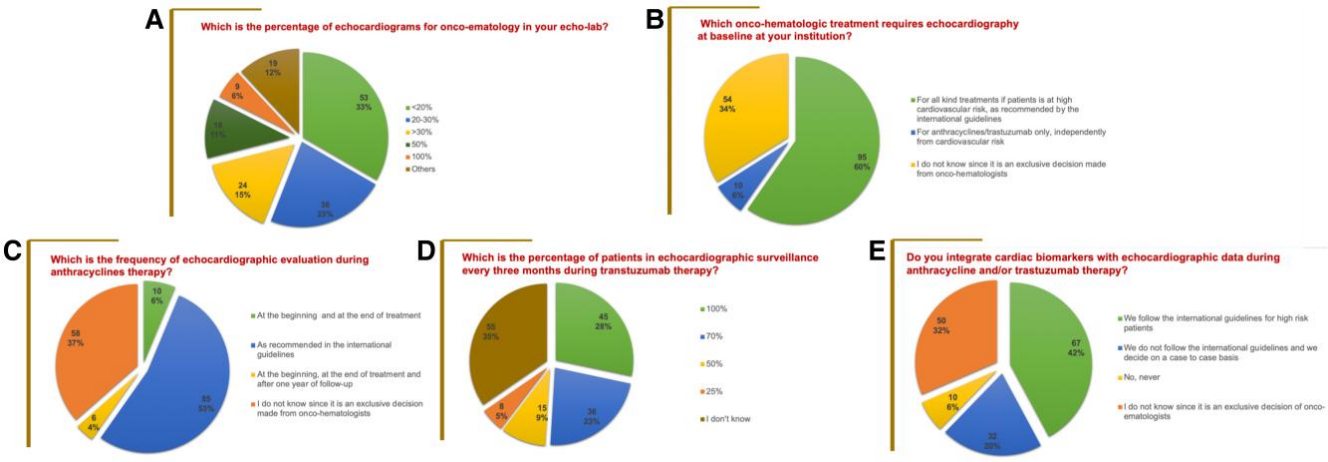
Pie charts of replies to survey items. (*A*) Percentage of echocardiograms for onco-haematology. (*B*) Treatment requiring echocardiography at baseline. (*C*) Frequency of echocardiographic surveillance during anthracycline treatment. (*D*) Frequency of echocardiographic surveillance during trastuzumab. (*E*) Integration of echocardiography with biomarkers during anthracycline/trastuzumab treatment.

Thirty per cent of respondents affirmed that echocardiographic activity in cancer patients accounted for >30% of total lab activity (*Figure 2A*). The most common TTE indication was the evaluation of LVEF (85%) before or during potentially cardiotoxic therapies. However, 34% of respondents stated they were unaware of which oncological/haematological therapy a baseline TTE required since the indication for TTE was entirely left to the oncologist’s discretion (*Figure 2B*).

### Adherence of participating centres to available cardio-oncology recommendations and use of advanced echocardiography parameters

During anthracycline therapy, more than half of responding centres (53%) declared that cardiologists routinely followed the ESC 2022 Cardio-Oncology guidelines for patients’ baseline risk stratification and surveillance. However, although participants affirmed acknowledged recommendations, 37% of them could not define the exact timing of surveillance because the re-evaluation scheduling was entrusted to oncologists. As far as surveillance during HER2 inhibitors is concerned, only 28% of centres declared to follow the quarterly frequency of echocardiographic controls indicated by the ESC 2022 Cardio-Oncology guidelines,^1^ and a non-negligible proportion of respondents (35%) could not define the best timing for re-evaluations in these patients (*Figure 2C* and *D*).

**Figure 3:**
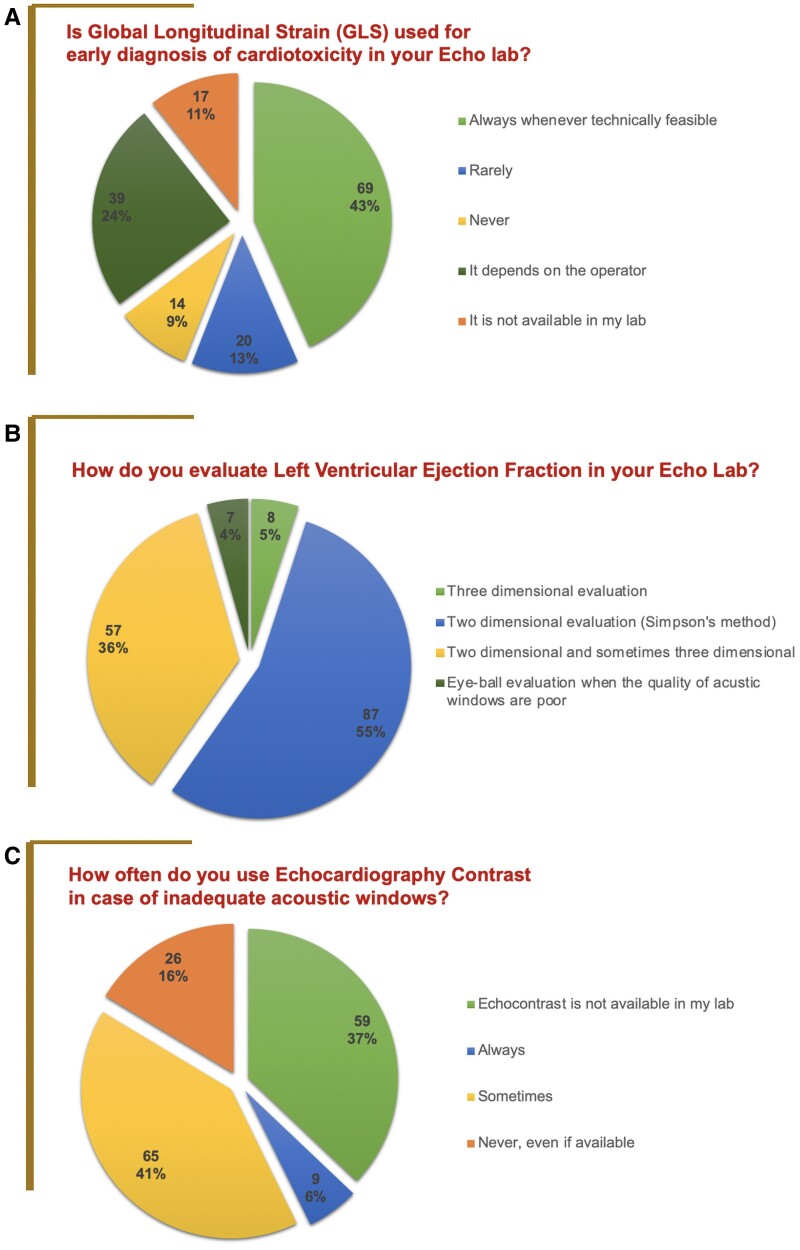
Pie charts of replies to the survey items. (*A*) The use of GLS in cardiotoxicity diagnosis. (*B*) Methods used for ejection fraction evaluation. (*C*) Use of contrast echocardiography.

Of interest, 42% of centres declared to closely observe recommendations from 2022 ESC guidelines on integrating serum biomarkers (e.g. troponin and natriuretic peptides) with TTE data during treatment with anthracyclines and/or trastuzumab for early cancer therapy-related cardiac dysfunction (CTRCD) detection. However, as many as 32% of participants could not answer because they affirmed that they were not involved in serum biomarkers’ application in clinical practice and left the decision to treating onco-haematologists (*Figure 2E*). Furthermore, when speckle tracking echocardiographic metrics were investigated, only 43% of participants followed recommendations and routinely used GLS, when technically feasible, to diagnose subclinical cardiotoxicity (*Figure 3A*). Furthermore, as many as 76% of respondents did not perform left atrial strain to corroborate the diagnosis of CTRCD.

Only 5% of centres consistently used 3D echocardiography for LVEF estimation; 36% of respondents used 3D TTE only occasionally (*Figure 3B*). Many centres assessed left ventricular systolic function using bidimensional echocardiography. In patients with inadequate acoustic windows, the majority (53%) of centres did not use UCA during TTE to optimize myocardial segment visualization. More than half (55%) of centres affirmed that they employ a subjective visual assessment of LVEF (*Figure 3C*).

### Subgroup analysis according to the presence of dedicated cardio-oncology units

We performed a pre-specified sub-analysis according to the presence (52 centres, 33%) or absence (107 centres, 77%) of dedicated cardiologists for echocardiographic surveillance of cancer patients. According to the presence or absence of a cardio-oncologist, the characteristics of centres are depicted in *Table 1*.

**Table 1:** Cardiology activity dedicated to cancer patients according to the presence or absence of specialized cardiologists.

	Dedicated cardiologists, *N* = 52	Non-dedicated cardiologists, *N* = 107	*P*
Northern regions, *N* (%)	19 (36.5)	65 (60.7)	*0.013*
Central regions, *N* (%)	15 (28.8)	16 (15.0)	
Southern regions, *N* (%)	18 (34.6)	26 (24.3)	
Type of hospital			
General hospital, *N* (%)	36 (69.3)	93 (86.9)	*<0*.*001*
Oncology hospital, *N* (%)	10 (19.2)	2 (1.9)	
Outpatient clinics, *N* (%)	6 (11.5)	12 (11.2)	
TTE in cancer patients, *N* (%)			*<0*.*001*
<20%	5 (9.6)	48 (44.9)	
20–50%	18 (34.6)	42 (39.3)	
>50%	29 (55.8)	17 (15.9)	
Type of request, *N* (%)			
Evaluation of EF	39 (75.0)	97 (90.7)	*0*.*008*
Symptoms of HF	5 (9.6)	1 (0.9)	
Other	8 (15.4)	9 (8.4)	
3D in the assessment of EF performed, *N* (%)	22 (42.3)	43 (40.2)	*0*.*799*
3D in the assessment of EF not performed, *N* (%)	30 (57.7)	64 (59.8)	
Use of GLS, *N* (%)			
Never	10 (19.2)	21 (19.6)	*0*.*052*
Rarely	8 (15.4)	12 (11.2)	
Always	28 (53.8)	41 (38.3)	
Depends on the operator’s skill	6 (11.5)	33 (30.8)	
Atrial strain performed, *N* (%)	11 (21.2)	54 (50.5)	
Atrial strain not performed, *N* (%)	41 (79.8)	53 (49.9)	*0*.*491*
UCAs performed, *N* (%)	20 (38.5)	28 (26.2)	*0*.*154*
UCAs not performed, *N* (%)	32 (61.5)	79 (73.8)	
Cancer therapy and TTE, *N* (%)			
Only anthracyclines and trastuzumab	5 (9.6)	5 (4.7)	*<0*.*001*
All therapy in patients with elevated CV risk	41 (78.8)	54 (50.5)	
Depends on the oncologist’s decision	6 (11.6)	48 (44.9)	
During anthracycline therapy, TTE performed, *N* (%)			*<0*.*001*
Start and end therapy	6 (5.8)	7 (6.5)	
Start and end therapy at 1 year	1 (1.9)	5 (4.7)	
According to ESC 2022-GGLL	42 (80.8)	43 (40.2)	
Depends on the oncologist’s decision	6 (11.5)	52 (48.6)	
During trastuzumab therapy, TTE is performed every 3 months, *N* (%)			*<0*.*001*
25%	3 (5.8)	5 (4.7)	
50%	4 (7.7)	11 (10.3)	
75%	15 (28.8)	21 (19.6)	
100%	28 (46.2)	21 (19.6)	
Don’t know the answer	6 (11.6)	49 (45.8)	
Use of biomarkers, *N* (%)			
Never	6 (5.8)	4 (3.7)	*<0*.*001*
Adjust as appropriate	14 (26.9)	18 (16.8)	
According to ESC 2022-GGLL, in patients with elevated CV risk	30 (57.7)	37 (34.6)	
Depends on the oncologist’s decision	2 (3.8)	48 (44.9)	

TTE, transthoracic echocardiography; EF, ejection fraction; HF, heart failure; 3D, three-dimensional echocardiography; GLS, global longitudinal strain; UCAs, ultrasound contrast agents; CV, cardiovascular; ESC 2022-GGLL, European Society of Cardiology Guidelines on cardio-oncology.

Centres with dedicated cardiologists were more frequently located in the central and southern regions (63%) compared to the northern regions of Italy (39%) (*P* = 0.01). Moreover, 90% (10 centres over 12) of Italian highly specialized oncology hospitals had cardio-oncologists on their team.

In centres without dedicated cardiologists, TTE in cancer patients was performed more rarely (<20% of the total volume of activity in 44.9% of these centres) compared to centres with a cardio-oncology service. In contrast, in these latter institutions, TTE in cancer patients represented >50% of the total activity volume, as recorded by 55.7% of respondents.

Of note, the presence of a dedicated cardiologist was not associated with a substantial difference in the use of advanced echocardiographic imaging methods.

When participants were asked about their relationships with oncologists regarding decision-sharing and cardiovascular surveillance strategies, it was necessary to note that the differences between centres with and without dedicated cardiologists were more apparent. Indeed, in approximately half of the centres without dedicated cardiologists, physicians performing TTE in cancer patients declared not to be aware of the management of eventual cardiovascular complications of cardiotoxic therapies, including the number of echocardiograms required, the use of cardiac biomarkers, and follow-up at the end of treatment (cancer survivors’ care).

## Discussion

It has been around a decade since the European Association of Cardiovascular Imaging (EACVI) and the American Society of Echocardiography released proposals for homogeneous application of echocardiography in cancer patients, especially for those undergoing cardiotoxic therapies.^16^ Afterward, position papers and guidelines were published to guide the appropriate management of adverse cardiac events and plan surveillance.^1^ However, real-world data on applying these recommendations in clinical practice are limited, and information on different aspects of echocardiography use in this setting is almost entirely missing.

The main results of our inter-society, nationwide survey involving 159 cardiology centres include the following: (i) echocardiography undoubtedly plays a major role in cancer patients’ management; (ii) there is considerable divergence among Italian regions and health organizations in terms of dedicated figures specialized in echocardiographic surveillance of oncologic patients; (iii) indications and scheduling for echocardiograms and serum biomarkers are essentially left to treating oncologists and haematologists; (iv) LVEF represents the most required information from TTE, often quantified subjectively, while GLS and other speckle tracking metrics are less frequently performed; and (v) the majority of respondents are unaware of guideline recommendations on appropriate surveillance strategies, even for therapies with well-established cardiotoxic effects (i.e. anthracyclines and/or trastuzumab).

Echocardiography represents the first-line cardiovascular imaging modality in patients undergoing chemotherapy. The present survey indicates that, in Italy, cardio-oncology activity heavily relies on echocardiography, mainly for LVEF estimation. However, a substantial divergence emerged between clinical practice and recommendations when we investigated participants’ knowledge levels regarding TTE indications and planning in oncologic patients. Indeed, in approximately one-third of participating centres, the cardiologist who performed an echocardiogram for a cancer patient was neither involved in the indication nor in scheduling the frequency of follow-up. This percentage was even higher in hospitals without a dedicated cardiologist with expertise in oncologic patient care. Our findings parallel those of an EACVI survey conducted in 2020 involving 104 centres from 35 countries, mainly in Europe. This survey showed that the care of patients with adverse cardiovascular effects from cancer therapies was provided by a dedicated cardio-oncology unit only in 14% of centres. In comparison, 42% of EACVI survey participants affirmed that they did not have a formalized cardio-oncology team but cardiologists with expertise in the field. At that time, 44% of centres declared no communication between oncologists and cardiologists when managing cardiovascular complications.^17^ Taken together, the real-world results from the EACVI and present ANMCO/SIECVI survey, even though performed pre and post the ESC cardio-oncology guideline release, showed, in the same way, the arduous journey to build multidisciplinary programmes focused on management and outcomes of cardiovascular complications in cancer patients.

Moreover, these results also highlight the lack of appropriate knowledge diffusion in this rising branch of cardiology, with a considerable proportion of cardiologists partially or entirely unaware of cardiotoxicity definitions and diagnosis modalities. As shown from our results, despite available recommendations stating to use GLS during surveillance and advising to preferably use 3D echocardiography for LVEF estimation to enhance the accuracy of heart function assessment and reduce inter-/intra-observer variability, advanced imaging methods still find little application in everyday practice. This may appear to go against the results of the EACVI survey,^17^ in which almost 90% of European responding centres declared access to 3D and speckle tracking echocardiography, suggesting the wide availability of these modalities. However, results should be put in the context of regional and national realities. In-depth, in our survey, LVEF was routinely assessed using 3D modality in 5% of respondents and only occasionally in 36% of cases; GLS was instead regularly used by 43% of centres. In Italy, the use of advanced echocardiography in cardio-oncology appears therefore low, but consistent with data coming from a recent SIECVI survey on the current national availability of advanced echocardiography imaging, which demonstrated that these modalities were frequently available only in centres with high-volume activity and employed only in selected cases.^18^

As advised by the recently published cardio-oncology Core Curriculum, echocardiography-trained cardiologists are essential team members of cardio-oncology services,^7^ and access to all echocardiographic parameters (both conventional and advanced) should be considered as a pre-requisite for cardio-oncology unit.^19^ On the other hand, it is known that the application of advanced imaging is subject to image quality. Cardiotoxicity monitoring with these metrics may be difficult when echocardiographic images are inadequate. In these cases, more than half of the responding centres estimated LVEF only visually. At the same time, UCA was not performed in 51% of centres, mainly because of the unavailability of contrast medium. This highlights the critical problem of reproducibility and reliability of any imaging modality, particularly TTE in cancer patients, where variations in cardiac function can influence decisions on oncologic treatments and cardioprotective strategy adoption. In this context, cardio-oncologists may again play a crucial role: cardiologists with proper expertise in cardiac imaging and management of chemotherapy-related cardiovascular complications, being in continuous collaboration with oncologists and haematologists, should be aware of patients’ baseline risk of CTRCD and should intercept eventual declines in cardiac function to establish an appropriate cardioprotective therapy, even when advanced imaging metrics are not available and taking advantage in this decision of information deriving from serum biomarkers.

Even though changes in GLS have been shown to predict LV dysfunction,^20^ its role in identifying patients who might benefit from the use of angiotensin-converting enzyme inhibitors or beta-blockers to prevent LVEF decline has been questioned in the low-risk population of the Strain Surveillance of Chemotherapy for Improving Cardiovascular Outcomes (SUCCOUR) trial.^21^ This could explain, at least in part, the lack of significant differences in the use of GLS when subgroup analysis was performed between centres with and without a dedicated cardio-oncology service, highlighting the restriction of actual GLS application to cases of borderline-normal LVEF.^22^ At the same time, it shows the precious role of dedicated cardio-oncologists who can integrate imaging biomarkers, even though limited, with judicious clinical assessment. When available, advanced imaging modalities, such as myocardial strain imaging, may offer a valuable opportunity to complement the clinician’s opinion with myocardial function estimation, particularly when this latter results in borderline-normal ranges, to improve detection of cardiotoxicity and mitigate cardiovascular morbidity.

The predictive role of cardiac biomarkers in cancer surveillance has been extensively investigated in clinical trials.^23^ As a result, guidelines recommend that troponins and natriuretic peptides be dosed serially after chemotherapy administration, according to CTRCD baseline risk, and, if abnormal, to evaluate cardioprotective therapy establishment. In our survey, 42% of centres declared to closely observe 2022 ESC guidelines regarding integrating serum biomarkers with TTE data during anthracycline and/or trastuzumab treatment. However, 32% of participants left it to treating onco-haematologists to decide how often to dose biomarkers without having a role in their interpretation. In these terms, differences became statistically significant when respondents were stratified according to the presence of a dedicated cardio-cardiologist in the institution. This again highlights the necessity of a cardio-oncology multidisciplinary team, able to decide when to perform cardiac biomarkers (according to the institution’s possibilities), to integrate results from echocardiography and electrocardiogram with eventually altered laboratory markers, and to modify patients’ care pathway, always keeping in mind the concept of ‘permissive cardiotoxicity’.^24^

Finally, a crucial point emerging from our data concerns dedicated education and commitment from scientific societies on cardio-oncology knowledge diffusion: growing clinical demand has generated the need for special training of physicians in this field, further reflected by the explosive research interest in the topic in the last few years. As a result, the spectrum of knowledge required for delivering a cardio-oncology service and ensuring effective cancer patients’ cardiovascular care now exceeds what is included in the curriculum for a general cardiologist, encompassing basics of cancer biology, anticancer therapies, pharmacological interactions, and protocols of cardiovascular assessment or surveillance during treatment and in survivors. Differences in managing cardiac events in this vulnerable population, compared with the general one, should also be known. This gap may be filled in the future through teaching courses focused on cardio-oncology during cardiology/oncology/haematology residency, dedicated congresses, and the development of certification programmes to improve the standards of cardiovascular care for cancer patients.^25^

### Limitations

While providing valuable insights, our survey faces limitations. The open-access nature of the survey may affect the quality of the data. However, the proposed questions have been carefully designed to be as straightforward and understandable as possible (see Supplementary data online, *File S1*). Additionally, considering the national nature of the survey, results may not be generalized to other countries in which practices and clinicians’ attitudes may differ. Furthermore, the ANMCO and SIECVI’s email lists were used to contact participants; these lists include the majority, but certainly not all, of the echocardiographic laboratories caring for cancer patients in Italy. Some centres not included in this list may also have high-volume activity and high-quality standards. However, although this survey might have underestimated the cardio-oncology activities in selected centres, it likely represents the quality standards and patterns of cardio-oncology practice in Italy.

## Conclusion

This inter-society Italian survey on cardiac imaging practice in oncologic patients reveals a significant gap between clinical practice and proposed standards by dedicated ESC guidelines. Therefore, large-scale, national, and international registries should be performed to better characterize the attitudes of echocardiographic laboratories towards cancer patients’ care pathways. Integrating imaging biomarkers among outcomes of oncology clinical trials and post-marketing surveillance studies also appears to be a rising necessity. They have recognized a notable lag phase between the release of recommendations and their application in clinical practice. Results from our survey call for dedicated education in cardio-oncology and homogeneous diffusion of specialized units caring for cardiac diseases in cancer patients.

## Supplementary Material

qyae081_Supplementary_Data

## Data Availability

The article’s data are provided by the Italian Society of Echocardiography and Cardiovascular Imaging (SIECVI) and ANMCO by permission. Data will be shared on request with the corresponding author with the authorization of the SIECVI and ANMCO.

## Lead author biography

Andrea Barbieri currently works as a senior cardiologist at Azienda Ospedaliero-Universitaria di Modena-Policlinico, Italy, where he is the Head of Echocardiography Laboratory since 2001. He is the author of 104 papers in peer-reviewed international journals and 99 abstracts in ISI journals and/or meeting proceedings, with an H-index 29 (Scopus), and a reviewer for several international journals. His main research interests are valvular heart disease, heart failure, left ventricular hypertrophy, and stress echocardiography. Of note, he participates to Mitral Regurgitation International Database (MIDA) international registry. He is part of the National Board of SIECVI (Società Italiana di Ecocardiografia e CardioVascular Imaging) from 2019.
