# Supplementary material for: Current use of echocardiography in cardio-oncology: nationwide real-world data from an ANMCO/SIECVI joint survey
Source: Eur Heart J Imaging Methods Pract. 2024 Aug 12;2(3):qyae081. doi: 10.1093/ehjimp/qyae081 (PMC11367962; doi:10.1093/ehjimp/qyae081)
Supplement: qyae081_Supplementary_Data [file qyae081_supplementary_data.docx]

The complete questionnaire for the joint ANMCO/SIECVI survey on cardio-oncology echocardiographic practice in Italy.

**In which structure do you work?**

- General hospitals with an oncology division
- Highly specialized oncology hospitals
- Outpatient clinic

**What percentage of echocardiograms do you perform for cancer patients in your practice?**

- <20%
- 20-30 %
- >30%
- 50%
- 100%

**Who performs echocardiography for cancer patients?**

- There is no dedicated cardiologist
- Cardiologists are dedicated exclusively to imaging service
- The cardio-oncologist carries out both clinical and instrumental evaluation

**Which question is most often specified in the request?**

- Left ventricular ejection fraction assessment
- Appearance of symptoms or signs of decompensation
- Other

**For which oncological treatments is a baseline echocardiogram required?**

- All treatments in patients at high cardiovascular risk, as provided for in the Guidelines
- Only for anthracyclines and/or trastuzumab, regardless of risk
- I don't know the answer (because it is an exclusive decision of the oncologists)

**How often do you perform an echocardiogram during anthracycline therapy?**

- At the beginning and the end of the treatment
- We follow the ESC 2022 guidelines: also, in the follow-up
- Start, end of treatment, one year after the end of treatment, then stop
- I don't know the answer (because it is an exclusive decision of the oncologists)

**In what percentage of patients are treated with trastuzumab do you respect the quarterly frequency of echocardiographic checks?**

- 100%
- 70%
- 50%
- 25%
- I cannot answer

**Do you use global longitudinal strain for early diagnosis of cardiotoxicity?**

- Always, if it is technically possible
- Rarely
- Never
- Depends on the operator on duty
- It is not available in my office

**Do you perform atrial strain?**

- Yes
- Yes, but only for research purposes
- No, even if available
- No, because not available

**Do you use a contrast medium for echocardiography in cases with an inadequate acoustic window?**

- Not available
- Always
- Sometimes
- Never, even if available

**How do you perform the left ventricular ejection fraction assessment for cancer patients?**

- With 3D
- With 2D biplane
- With 2D and sometimes 3D
- Visual subjective assessment if the acoustic window is poor

**Do you integrate biomarkers with echocardiographic data during anthracyclines and/or trastuzumab treatment?**

- We faithfully follow guidelines for high-risk patients
- We do not follow the guidelines, and we adjust as appropriate
- Never
- I don't know the answer (because the cardiologist is not involved in the evaluation with biomarkers)
